# Is It a College?

**Published:** 1881-02

**Authors:** 


### EDITORIAL.

### IS IT A COLLEGE?

During the last two or three years there has been some agitation and excitement on the subject of "Bogus Colleges" and "Fraudulent Diplomas." All are familiar with the exposure and suppression of the notorious Buchanan and his gang at Philadelphia. The continuance of that institution, or rather institutions, for many years, in the nefarious business, is a strong evidence of the long suffering endurance of an outraged public. But there are strong indications that such traffic cannot be continued with impunity.

If the supply in this business is regulated by demand, then is society in a sad plight indeed, and a speedy remedy should be applied wherever the abuse is found to exist. It is publicly stated that such an institution has recently been found in Boston. This, and the one at Philadelphia, issued any and every sort of diploma that might be called for.

Just now appears above the surface the head of another institution, that, so far as developments are seen, is another of the same sort, only limited to dentistry. This makes its appearance well up in the Northwest. In Delevan, Walworth County, Wisconsin, is the "Wisconsin Dental College." In the little circular is contained the following information, viz.:

"This College is a regular organized corporation under the laws of Wisconsin and will be conducted with ability and integrity.

The requirements for graduation relating to age and to practical demonstration of skill at the operating chair, and in the laboratory, are the same that are usually demanded by all reputable Dental Schools, except in this institution students receive Diplomas and degree D. D. S. for what they know and can do, and not for the number of terms spent at the College.

"FACULTY. D. B. Devendorf, M. D., Professor of Anatomy and Surgery. Dr. John Morrison, Professor of Operative Dentistry. Dr. George Morrison, Professor of Mechanical Dentistry.

"FEES. Matriculation, $5.00. Tickets for One Term, $60.00. Diploma, $25.00. For additional information address

DR. GEO. MORRISON, Pres.,
Delevan, Wis."

Now that something else than that indicated in the above is being done, is pretty clearly shown by the following, to which the special attention of every reader should be given.

In order that there may be no misapprehension, a letter and statement sent out by this institution is given in full, with names, places and date. They are as follows:

"WISCONSIN DENTAL COLLEGE, Incorporated under the Laws of Wisconsin.

D. B. DEVENDORF, M. D., Professor of Anatomy and Surgery.
Dr. JOHN MORRISON, Professor of Operative Dentistry.
Dr. GEORGE MORRISON, Professor of Mechanical Dentistry.

Delavan, Wis., Dec. 31, 1880.

DR. WM. VAN ANTWERP:

Dear Sir: By mail I send you the Wisconsin Dental College announcement. Should you conclude to take a full or part of the term, we will give you practical instructions, which will be to your advantage; however, if you do not desire a College course, I take the liberty to make you this offer. Fill the blanks in the enclosed printed statement and return. If satisfactory to the Faculty, will send you in a cylinder box by express C. O. D. $12.00, an elegant *Honorary* Diploma and Degree D. D. S. (Doctor of Dental Surgery) with your name artistically hand printed. This Diploma is 22 x 17 inches, elaborately engraved on *parchment*, with signatures of the Faculty and College Seal. Should you accept of this compliment from the Wisconsin Dental College, we shall expect your influence by way of assisting us to students in the future.

Respectfully, your servant,
GEO. MORRISON, Pres't."

"..................................................188

This statement is made to the Faculty of the Wisconsin Dental College, located at Delavan, Walworth Co., Wisconsin, for the purpose of procuring an Honorary Diploma and Degree D. D. S. (Doctor of Dental Surgery.)

I am a regular practicing Dentist.

I am a resident of.......................................age,....................years.
Have practiced Dentistry.................................years.

Signature.................................................
Witness.................................................."

I think no one will ask for additional information, at least, so far as the meaning of these documents is concerned. (Dr. Wm. Van Antwerp resides at Mt. Sterling, Ky., to which place the above was sent by mail.) This heap is all *cat;* there is not even a little meal sprinkled over it; and if any is deceived by it, it will be because he does not know a cat when he sees it.

I have learned of quite a number of other parties, nearly all in Kentucky, to whom similar documents have been sent. What has Kentucky done to be thus maltreated?

It is suggested that the dental profession of Wisconsin look after its College, and things.

